# ABO, Lewis, secretor and non-secretor phenotypes in patients infected or uninfected by the *Helicobacter pylori* bacillus

**DOI:** 10.1590/S1516-31802002000200006

**Published:** 2002-03-02

**Authors:** Luiz Carlos de Mattos, Juliana Rodrigues Cintra, Fábio Eduardo Sanches, Rita de Cássia Martins Alves da Silva, Milton Artur Ruiz, Haroldo Wilson Moreira

**Keywords:** Blood groups, ABO, Lewis, Secretor phenotype, Non-secretor phenotype, *Helicobacter pylori*, Grupos sangüíneos, Sistema ABO, Sistema Lewis, Fenótipo secretor, Fenótipo não-secretor, *Helicobacter pylori*

## Abstract

**CONTEXT:**

Epidemiologicalstudies have demonstrated higher frequencies of the O blood group and the non-secretor phenotype of ABH antigens among patients suffering from peptic ulcers. Since *Helicobacter pylori* has been established as the main etiological factor in this disease, controversies about the associations of the ABO and Lewis blood group phenotypes and secretor and non-secretor phenotypes in relation to susceptibility towards infection by this bacillus have been presented.

**OBJECTIVE:**

To verify the frequencies of ABO, Lewis blood group phenotypes, secretor and non-secretor phenotypes in patients infected or uninfected by *H. pylori*.

**DESIGN:**

Cross-sectional study.

**SETTING:**

Outpatient clinic.

**PARTICIPANTS:**

One hundred and twenty patients with dyspeptic symptoms who underwent endoscopy.

**MAIN MEASUREMENTS:**

ABO and Lewis blood group phenotypes were determined by a standard hemagglutination test and the secretor and non- secretor phenotypes were evaluated by saliva samples using the inhibitor hemagglutination test.

**RESULTS:**

The diagnosis of infection, made via breath and urea tests and confirmed using polymerase chain reaction (PCR) in gastric biopsy fragments, showed the presence of H. pylori in 61.7% of the patients and absence in 38.3%. The differences between the frequencies of the ABO blood group phenotypes among infected (A 27.0%; B 12.2%; AB 4.0% and O 56.8%) and uninfected patients (A 58.7%; B 13.0%; AB 4.3% and O 24.0%) were significant. The Lewis blood type, secretor and non-secretor phenotypes showed homogeneous distribution between the groups of patients analyzed.

**CONCLUSIONS:**

Our results suggest that the infection of *H. pylori* can be related to ABO blood groups but not to the Lewis blood group nor to secretor and non-secretor phenotypes.

## RESUMO

**CONTEXTO:**

Estudos epidemiológicos evidenciaram alta freqüência do tipo O e do fenótipo não-secretor dos antígenos ABH em pacientes com úlceras pépticas. Desde que o *Helicobacter pylori* foi estabelecido como o principal agente etiológico destas doenças, controvérsias sobre as associações dos grupos sangüíneos ABO, Lewis e dos fenótipos secretor e não-secretor com a suscetibilidade à infecção por esse bacilo foram apresentadas.

**OBJETIVO:**

Verificar as freqüências dos fenótipos ABO, Lewis, secretor e não-secretor em pacientes infectados ou não infectados pelo H. pylori.

**TIPO DE ESTUDO:**

Estudo transversal.

**LOCAL:**

Ambulatório de Gastroenterologia do Hospital de Base de São José do Rio Preto e Laboratório de Imunogenética Molecular do Hemocentro de São José do Rio Preto.

**PARTICIPANTES:**

Foram avaliados 120 pacientes com sintomas de dispepsia submetidos a endoscopia digestiva alta.

**VARIÁVEIS ESTUDADAS:**

Fenótipos eritrocitários ABO e Lewis determinados pelos métodos de hemaglutinação padronizados e fenótipos secretor e não-secretor pelo método de inibição de hemaglutinação.

**RESULTADOS:**

O diagnóstico de infecção e não- infecção, realizado pelos testes respiratório e da urease, e confirmado pelo método da reação de polimerase em cadeia (PCR) em fragmentos de biópsias gástricas demonstrou a presença do *H. pylori* em 61,7% dos pacientes e ausência em 38,3%. As diferenças nas freqüências dos grupos sangüíneos ABO entre os pacientes infectados (A 27,0%; B 12,2%; AB 4,0%; O 56,8%) e não-infectados (A 58,7%; B 13,0%; AB 4,3%; O 24,0%) foram significantes. Os tipos sangüíneos Lewis e os fenótipos secretor e não-secretor mostraram distribuição semelhante entre os grupos de pacientes analisados.

**CONCLUSÕES:**

Os resultados sugerem que a infecção pelo *H. pylori* parece ser influenciada pelos fenótipos ABO, mas não pelos fenótipos Lewis, secretor e não-secretor.

## INTRODUCTION

The ABO system is the most investigated erythrocyte antigen system for all populations and, due to the ease of identifying its phenotypes, it has been used as a genetic marker in studies of associations with infectious and non-infectious diseases.^1,2^ On the other hand, the Lewis blood group system and the secretor and non-secretor phenotypes of ABH antigens have been less intensively studied.^1^

A compilation by Lima et al. of results from different studies among Brazilians showed that the frequencies of the ABO and Lewis blood group systems and the secretor and non-secretor phenotypes are similar to those observed in European populations. ^3^ Recently, Mattos et al. demonstrated that the ABO blood groups among Brazilians are highly polymorphic when analyzed by molecular methods.^4^

Among the first epidemiological studies to establish associations between blood groups and diseases, there were some demonstrations of high frequencies of the O blood group and non-secretor phenotype of ABH antigens among patients suffering from peptic ulcers.^5,6^

It was later shown that the *Helicobacter pylori* bacillus is the main etiologic agent associated with gastric ulceration, being present in more than 80% of patients with this disease.^7,8^ Subsequently, it was demonstrated that infection by this bacillus affects more than 90% of the adult population around the world, especially in underdeveloped countries.^9^ In Brazil, investigations of the presence of *H. pylori* among children, youths and blood donors have demonstrated that the frequency of this infection ranges from 34% to 66%.^10,11^

In the early 90s, Borén et al. reported that this bacillus chooses to attach itself to the Lewis b antigen (Le^b^), which is rich in fucose and is expressed on the surface of the epithelial cells of the gastric mucosa.^12^ With this observation, the authors tried to establish connections between the associations of the ABO blood groups and the secretor phenotypes of ABH antigens with peptic ulcers that had been observed in previous decades.

The ABH and Lewis antigens on the gastric and duodenal mucosae are synthesized through a specific glycosyl transferase, which incorporates molecules of fucose in common type I oligosaccharide precursor.^13,14^ The O and Le(a-b+) phenotypes express a greater quantity of these fucosylated antigens in comparison with other groups, and Borén et al. believed that this difference predisposed these carriers to *H. pylori* infection.^12^^,^^15^

Various studies have evaluated the associations of the ABO and Lewis blood groups and the secretor and non-secretor phenotypes with *H. pylori* infection. Hooke- Nikanne et al. did not find an association between the secretor phenotype and positive serology for this bacillus among 271 blood donors.^16^ Clyne & Drum observed that the Le^b^ antigen expression did not influence the adherence of *H. pylori* to the gastric epithe- lium.^17^ On the other hand, Alkout et al. showed that the O blood group had great susceptibility towards peptic ulcers and that the H and Le^b^ antigens had an influence on *H. pylori* infection.^18^ Lin et al. demonstrated a high frequency of infection with this bacteria in 90.3% of O blood group patients suffering from gastroduodenal diseases.^19^

These data show that the associations of ABO, Lewis, secretor and non-secretor phenotypes with *H. pylori* infection are controversial because of the discordance between clinical observations and laboratory evidence.^12,15-19^

The aim of this study was to verify the frequencies of ABO, Lewis, secretor and non- secretor phenotypes in patients with symptoms of dyspepsia who underwent upper gastrointestinal endoscopy and to cross-check whether they were infected by *H. pylori* or not.

## METHOD

One hundred and twenty adult patients who were seen over a period of one year in the Department of Endoscopy and/or the Clinical Gastroenterology outpatient service of the Medical School (FAMERP) were evaluated. Patients were included in the study if they had symptoms of dyspepsia and if upper gastrointestinal endoscopy was indicated. This group included sufferers of gastric and duodenal ulcers.

The study had prior approval from the Research Ethics Committee of the Institution (case 4657/97) and informed consent was obtained from all the participants.

Patients who were less than 18 years old or pregnant, those who had gastrointestinal tract hemorrhage or acute gastritis, and those who had used a proton-pump inhibitor in the previous week or had used an H2 receptor antagonist in the previous 24 hours, were excluded from the study.

A sample of 5 ml of peripheral blood was obtained using EDTA to type the ABO and Lewis blood groups and 2 ml of saliva was collected for the determination of secretor and non-secretor phenotypes.

ABO blood groups were determined using standardized hemagglutination methods^20^ and the Lewis phenotypes were ascertained using the gel method.^21^ Secretor and non-secretor phenotypes were identified using the hemagglutination inhibition test.^22^ Investigation of *H. pylori* was done in a routine manner by a specialized gastroenterology laboratory using urea breath and urease tests. In all cases, whether positive, negative or discordant, the presence of the infection was confirmed using the polymerase chain reaction (PCR), in accordance with the protocol of Valentine et al.^23^ The χ^2^ test and dependence analysis were used to calculate associations among the results.

## RESULTS

One hundred and twenty patients, both male and female, including Caucasians and non-Caucasians, with a mean age of 42 years (ranging from 18 to 74 years old), were assessed and divided into groups infected and uninfected by *H. pylori*.

Of the 120 patients enrolled, 40.8% (49/ 120) were male and 59.2% (71/120) were female; 77.5% (93/120) were Caucasians and 22.5% (27/120) were non-Caucasians. *H. pylori* infection was present in 61.7% (74/120) and absent in 38.3% (46/120) of the patients. No significant differences were observed when comparing gender and racial group with *H. pylori* infection.

Table 1 shows the characteristics of infected and uninfected patients. Analysis of the whole data set demonstrated that the distribution of the ABO blood groups among the patients, independent of infection by *H. pylori*, followed the same proportions as the blood groups found in the general Brazilian population.^3,4^

**Table 1:** Characteristics of the patients infected or uninfected by *H. pylori*

Characteristics	Uninfected		Infected		χ^2^
Sex (M/F)	16/30		33/41		NS
Mean age	42.3		43.5		NS
	**N**	**%**	**N**	**%**	
ABO phenotypes					
O	11	24.0	42	56.8	S
A	27	58.7	20	27.0	S
B	6	13.0	9	12.2	NS
AB	2	4.3	3	4.0	NS
Lewis phenotypes					
Le(a+b-)	12	26.1	11	14.9	NS
Le(a-b+)	28	60.9	45	60.8	NS
Le(a-b-)	6	13.0	18	24.3	NS
Secretion phenotypes					
Secretor	35	76.1	60	81.1	NS
Non-secretor	11	23.9	14	18.9	NS
Total	46	38.3	74	61.7	

S = significant; NS = not significant.

When the frequencies of these phenotypes were analyzed separately in accordance with infection and non-infection, it was possible to verify that the O and A blood groups were distinct. Of the 74 infected patients, 56.8% (42/74) were type O and 27% (20/74) were type A; of the 46 uninfected patients, only 24% (11/46) were type O and 58.7% (27/ 46) were type A. The χ^2^ test demonstrated that the difference observed between the higher prevalence of type O in the infected group and the higher prevalence of blood group A in the uninfected group is significant, giving a p-value of 0.003. Dependence analysis applied to these results shows that there is an association between the blood group and infection by *H. pylori*, in which type O has a greater tendency towards infection and type A to non-infection.

The distribution of the Lewis, secretor and non-secretor phenotypes did not exhibit significant differences between infected and uninfected patients.

## DISCUSSION

Studies of associations between blood groups and diseases have interested researchers since the 1950s, when the first results showing a high prevalence of the O blood group among patients with peptic ulcer disease were published.^5,6^

These associations remained relatively unstudied until Borén et al. suggested that the Le^b^ antigen of the Lewis blood group system acts as a receptor for *H. pylori* on the gastric mucosa.^12^ The Le^b^ antigen is a oligosaccharide rich in fucose molecules and is expressed in greater quantities in the O blood group.^13,14^ Borén et al. investigated the hypothesis that, due to the presence of fucose molecules in the Le^b^ antigen, the carriers of the O blood group would have a greater tendency towards infection by *H. pylori*.^12,15^ These researchers believed that this host characteristic justified the observation made during previous decades that persons with blood group O have a greater tendency towards peptic ulcers in comparison with those with the A, B and AB blood groups, which continues to be reported in current publications.^24,25^

With the demonstration that *H. pylori* is the agent in the majority of cases of peptic ulcer disease, several studies have tried to establish a relationship between the presence of this bacillus and the ABO and Lewis blood groups and the secretor and non-secretor phenotypes.^12,15-19^

The results of this current study show a strong association between the O blood group and infection caused by *H. pylori*, which is reinforced by data obtained from other scientific papers.^18,19^ Significant differences observed in the distribution of the frequencies of infected patients in comparison with uninfected patients support the epidemiological view of the greater susceptibility of blood group O to infection by *H. pylori*.

These observations support the conclusions of Alkout et al., who demonstrated that the H antigen represents an important receptor expressed in the gastroduodenal mucosa cells to which *H. pylori* adheres.^18^ Mollicone et al. showed that the H antigen expression in the duodenal mucosa is controlled by *FUT1(H)* using type II oligosaccharide precursor.^26^ As this fucosylated antigen is not modified to A or B antigens in the O blood group, its greatest potential expression can be a important factor in establishing a connection between this blood group, the *H. pylori* infection and the diseases resulting from its presence, independent of Lewis phenotypes and the secretory or non- secretory condition of the ABH antigens.

Our results disagree with some previous reports in which it was demonstrated that the O blood group did not represent a risk factor for *H. pylori* infection.^16,27-29^ In these studies, the authors used other methods to detect infection by *H. pylori*, including ELISA,^16,28,29^ urease^27,28^ and histological^27,28^ tests. In our work, the confirmation of infection by this pathogen was based on PCR tests. There is strong evidence that other tests employed in the diagnosis of *H. pylori* infection differ in specificity and sensitivity when compared to the PCR.^23,30-34^ Atherton et al. used PCR to show the presence of *H. pylori* in patients treated with amoxicillin whose urease and histological test results were negative.^34^ This characteristic of PCR may have an influence on the different infection frequencies observed within distinct populations, by improving the diagnosis even in cases where there is low bacterial density on the gastric mucosa.^30,31,33^

Variations in the percentages between infected and uninfected patients, in relation to gender, Lewis phenotypes, secretor and non- secretor phenotypes did not result in significant differences. However, we observed an elevated frequency of the Le(a-b-) phenotype among infected patients. Our observations do not support the hypothesis that patients with the Le(a-b+) phenotype present a higher susceptibility to *H. pylori* infection.

## CONCLUSIONS

Our results suggest that *H. pylori* infection is strongly associated with the O blood group, which is in agreement with other published data, but there is no association with the Lewis, secretor and non-secretor phenotypes. Nevertheless, the high frequency of the O blood group among patients infected by this bacillus and in those with gastroduodenal diseases caused by the presence of *H. pylori* remains unexplained.
